# Algae extracts and methyl jasmonate anti-cancer activities in prostate cancer: choreographers of ‘the dance macabre’

**DOI:** 10.1186/1475-2867-12-50

**Published:** 2012-11-26

**Authors:** Ammad Ahmad Farooqi, Ghazala Butt, Zubia Razzaq

**Affiliations:** 1 Laboratory For Translational Oncology and Personalized Medicine, Rashid Latif Medical College, 35 Km Ferozepur Road, Lahore, Pakistan; 2 Phycology Research Laboratory, Department of Botany, GCU, Lahore, Pakistan; 3 Department of Physiology, Rawal Institute of Health Sciences, Lehtrar Road, Islamabad, Pakistan

## Abstract

There is an overwhelmingly increasing trend of analysis of naturally occurring ingredients in treatment of prostate cancer. Substantial fraction of information has been added that highlights activity at various levels and steps of deregulated cellular proliferation, metastasis and apoptosis. Among such ingredients, algae extracts and jasmonates are documented to have anti-cancer activity in vitro and in vivo and induce growth inhibition in cancer cells, while leaving the non-transformed cells intact. In this short review we outline systematically, how these ingredients predispose prostate cancer cells to undergo apoptosis.

## Introduction

Scientists worldwide are making efforts to design targeted therapies that can kill cancer cells without harming normal cells. Such therapies primarily rely on understanding of how and by what mean cancer cells differ from normal non-transformed cells. It is comprehensible that cancer cells do not resemble normal cells in terms of morphology and behavior. Confluence of information suggests that bioactive components extracted from algae, as well as methyl jasmonate (a natural compound belongs to the jasmonates family of plant stress hormones), seem to have anti-cancer activities through multiple mechanisms of action, including inhibition of cancer-cell growth and of invasion and metastasis, and through the promotion of apoptosis of cancerous cells. Considerable emerging evidence supports the inhibitory actions of bioactive components of algae and methyl jasmonate on prostate cancer. The purpose of this mini-review is to bring to limelight the findings from recent research regarding the potential effects and mechanisms of action of bioactive components of algae and methyl jasmonate on cancer. Finally, we highlight the research efforts that need to be made to facilitate the optimal development of algae derived bioactive ingredients.

### Apoptosis

Cell death pathway is subdivided into two categories namely extrinsic pathway and intrinsic pathway and former is initiated by binding of the extracellular ligands, TNF (tumour necrosis factor-) or FAS (apoptosis antigen-1) ligand (FASL), to death receptors TNFR1 (TNF-receptor1) and FAS. The resulting heteromeric complex activates procaspase 8, that mediates apoptosis through caspase 3 or alternatively another pathway intrinsic pathway is opted that initiates via cleavage of BID to tBID (truncated BID). FAS-mediated death occurs through a FADD-mediated activation of procaspase 8. Membrane permeabilization is an important step in intrinsic pathway and is mediated by BH3-only proteins, which sequester and restore the activity of BAX. It is notable that BAX is switched off by BCL2 thus inhibiting mitochondrial BAX translocation and the release of cytochrome c (Cyto c), SMAC/DIABLO, AIF and HTRA2/OMI. However activation of BAX facilitates the release of proteins from mitochondrion and cytochrome c interacts with APAF1 to recruit and activate caspase 9, forming the apoptosome, which activates the downstream executioner caspases 3 and 7. There is a push and pull between pro-apoptotic and anti-apoptotic proteins as SMAC/DIABLO sequesters and inhibits IAP proteins. Cell uses its hidden modulators to counteract antiapoptotic proteins (Farooqi et al., [1]; Farooqi et al., [2]).

Contemporary studies provide wealth of evidence that death receptors are signaling members of the tumour-necrosis factor receptor (TNFR) superfamily and decoy receptors, by contrast, are a non-signalling subset of the TNFR superfamily that abrogate death receptor mediated signaling cascade. Although there are some cells type specific studies that demonstrate potential of algal extracts in re-sensitizing resistant cancer cells to apoptosis via activation of death receptors (or block decoy receptors). Siphonaxanthin, a marine carotenoid from green algae has recently been demonstrated to decrease expression of Bcl-2 and remarkably enhanced activation of caspase-3 alongwith up-regulated expression of GADD45α and DR5 in human leukemia (HL-60) cells (Ganesan et al., [3]). It is also encouraging to note that there was a decline in levels of the X-linked inhibitor of apoptosis protein and survivin in the fucoidan-treated cells. Another study provided a multistep regulation of cell death pathway via fucoidan and it was shown that there was enhanced mitochondrial membrane permeability, as well as the cytochrome c and Smac/Diablo release from the mitochondria. Bak and truncated Bid proteins were raised in fucoidan treated cells, but a decline was noted in the levels of Mcl-1. Additionally, fucoidan increased the levels of the TRAIL, Fas and death receptor 5 proteins Kim et al., [4].

In the following segment we focus on how research with emphasis on algal extracts and methyl jasmonates roleplay in induction of apoptosis is sequentially and progressively maturing. We have developed a roadmap of research from key discoveries in fundamental biology to potential therapeutic applications.

### Algae

Cellular and molecular studies have shown algae derived ingredients to be potent naturally occurring anticancer compounds and have been suggested to prevent carcinogenesis.

Fucoxanthin is a marine carotenoid found in brown algae and is documented to display remarkable anticancer activity. Fucoxanthin suppressed the growth of LNCap prostate cancer cells in a concentration-dependent manner along with the induction of GADD45A expression and G(1) cell cycle arrest, but not apoptosis. Fucoxanthin activated c-Jun N-terminal kinase (SAPK/JNK), whereas the targeted inhibition of SAPK/JNK repressed the induction of G(1) arrest and GADD45A expression by fucoxanthin (Satomi et al., [5]). Another study had demonstrated notable efficacy of fucoxanthin metabolites (dietary fucoxanthin is hydrolyzed into fucoxanthinol) on the proliferation of PC-3 human prostate cancer cells (Asai et al., [6]; Ishikawa et al., [7]). Fucoxanthin was effective against various prostate cancer cell lines like PC-3, DU 145and LNCaP (Kotake-Nara et al., [8]).

Benzochromenone is a pigment obtained from an active extract of the marine crinoids *Comantheria rotula* using Bioassay-guided fractionation and significantly inhibited hypoxia inducing factor (HIF) activity, however vascular endothelial growth factor (VEGF), the target gene of HIF was not considerably repressed. Nonetheless these crinoids pigments differentially suppressed the growth of certain tumor cell lines Dai et al., [9]. Sodwanone a triterpenoid from a South African species of the marine sponge was also tested to be effective in inhibiting HIF-1 activation in PC-3 prostate tumor cells (Dai et al., [10]).

14-keto-stypodiol diacetate is a drug extracted from the algae *Stypopodium flabelliforme* and was indicated to disrupt microtubular organization and also inhibited cellular proliferation in DU-145 human prostatic cells (Depix et al., [11]). Glycoprotein of Capsosiphon fulvescens, a green sea algae induces apoptosis in human gastric cancer (AGS) cells Kim et al., [12].Fucose-containing sulfated polysaccharides (FCSPs) extracted from brown macro-algae are documented to induce apoptosis Ale et al., [13]. Studies have been performed that reveal the fact that Quinones and halogenated monoterpenes of algal origin are effective in inducing apoptosis in breast cancer cells in vitro de la Mare et al., [14].

Recently, efficacy of meroditerpenoids isolated from the brown alga Stypopodium flabelliforme were tested and it was demonstrated that these meroditerpenoids showed considerable activity against different cancer cell lines Pereira et al., [15].Yessotoxins obtained from red-tide algae, Protoceratium reticulatum are also effective in terms of induction of apoptosis Pang et al., [16]. Elatol, a compound (sesquiterpene) isolated from algae Laurencia microcladia displayed a remarkable reduction in tumour growth in C57BL6 mice (Campos et al., [17]). Bis(2,3-dibromo-4,5-dihydroxybenzyl) ether (BDDE) is a marine bromophenol compound derived from marine algae has recently been tested for apoptotic activity in K562 cells and is suggested to have potent activity (Liu et al., [18]). Mineral-rich extract derived from the red marine algae, *Lithothamnion calcareum* is effective in suppressing growth and inducing differentiation of colon carcinoma cells Aslam et al., [19].

*Sargassum siliquastrum* derived sargachromanol E (SE) is a chromene and has growth inhibitory effects on HL-60 cells in a concentration-dependent manner. The compound was also noted to induce apoptosis via caspase 3 activation (Heo et al., [20]). Polysaccharides extracted from the brown alga, *Sargassum latifolium* have considerable anticancer activity against leukemia Gamal-Eldeen et al., [21]. Fucoxanthin has also been tested for re-sensitizing the cells to drugs and findings indicated that the compound overcome drug resistance. The sensitization was restored by inhibition of drug resistance associated genes (rifampin-induced CYP3A4 and MDR1) expression in HepG2 hepatoma cells Liu et al., [22].

Triprenylated toluquinones and toluhydroquinones isolated from the *Arminacean nudibranch Leminda millecra* are also reported to induce ROS mediated apoptosis in esophageal cancer cells Whibley et al., [23].Contrary to previously described algae derived components which induce apoptosis, diphlorethohydroxycarmalol (DPHC), isolated from the brown algae *Ishige okamurae*, reduces the ROS levels and consequently reduced apoptosis in cells exposed to radiations (Ahn et al., [24]).

### Radioprotective role of algal extracts

In accordance with same concept, diphlorethohydroxycarmalol (DPHC), isolated from the brown algae Ishige okamurae, notably reduced the level of radiation-induced intracellular ROS and protected cells from undergoing cell death in cultured Chinese hamster lung fibroblast (V79-4) cells Ahn et al., [24].

### Jasmonates

Continuous research has offered better understanding of the fundamental processes involved in biosynthesis and the regulation of Jasmonic acid (JA) and its methyl ester, methyl jasmonate (MeJA), which are naturally occurring growth regulators found in higher plants. JA was first isolated from culture filtrates of the fungus Lasiodiplodia theobromae Griff. & Maubl., and MeJA was described as a component of the essential oils of Jasminum grandiforum L. and Rosmanus offincinalis L (Hu et al., [25]) and recently, these agents have received renewed consideration owing to reported anticancer properties. Accumulated evidence from experimental studies suggested that the structures of JA and MeJA were similar to mammalian eicosanoids, which were potent modulators of smooth muscle contraction and inflammatory responses. Various studies verified the fact that both eicosanoids and JA were derived from lipids in lipoxygenase-dependent pathways (Schaller and Stintzi, [26]).

Keeping in view the fact that Jasmonates have started to receive attention, several of the approaches based on targeting core components of the cell death machinery for cancer therapy are reviewed here.

It is worth mentioning that methyl jasmonate (MJ) and cis-jasmone (CJ) are documented to induce inhibition of growth in hormone independent prostate cancer cell lines, PC-3 and DU-145 (Yeruva et al., [27]). One study has indicated that overexpressed inhibitors of apoptosis (IAP) proteins in cancer cells often dampen the pro-apoptotic activity of methyl jasmonates. However introduction of IAP antagonists like SmacN7, potentiates MJ mediated apoptosis (Jiang et al., [28]).

Methyl jasmonate mediated apoptosis in A549 human lung adenocarcinoma cells was induced through activation of Bax and caspase-3 via reactive oxygen species production (ROS) (Kim et al., [29]). It is also attention-grabbing to note that methyl jasmonate suppresses the radiation-induced Bcl-2 expression and enhances the radiation sensitivity of prostate cancer cells (Ezekwudo et al., [30]).

Emerging findings provide ample evidence that prostate cancer cells constitutively generate 5-lipoxygenase (5-LOX) metabolites from arachidonic acid, and targeted inhibition of 5-LOX inhibits generation of 5-LOX metabolites and promotes cell death. MJ notably repressed uncontrolled proliferation of prostate cancer cells and showed specific interaction with 5-LOX enzyme pathway (Ezekwudo et al., [31]).

### Jasmonates and chemotherapeutic drugs

MJ displayed remarkably enhanced efficacy in combination with chemotherapeutic drugs in inducing death of several types of carcinoma cells (Heyfets et al., [32]). Various lines of evidence indicate that MJ can be added also to conventional X-ray and cisplatin therapeutic approaches to substantially improve their cytotoxic effect whereas lowering the effective dose (Milrot et al., [33]). Recently, a research group reported a new jasmonic acid stereoisomeric derivative that had remarkable potential to induce apoptosis in prostate cancer cells. It is worth explaining that ROS generation is a major mechanism that is opted to induce apoptosis (Russo et al., [34]). It has lately been explored that Perillyl alcohol (POH) and MJ treatment activated TNFR1 and response was increased by the addition of cisplatin (Yeruva et al., 2010[35]).

### Jasmonates and TRAIL join hands

**Figure 1:**
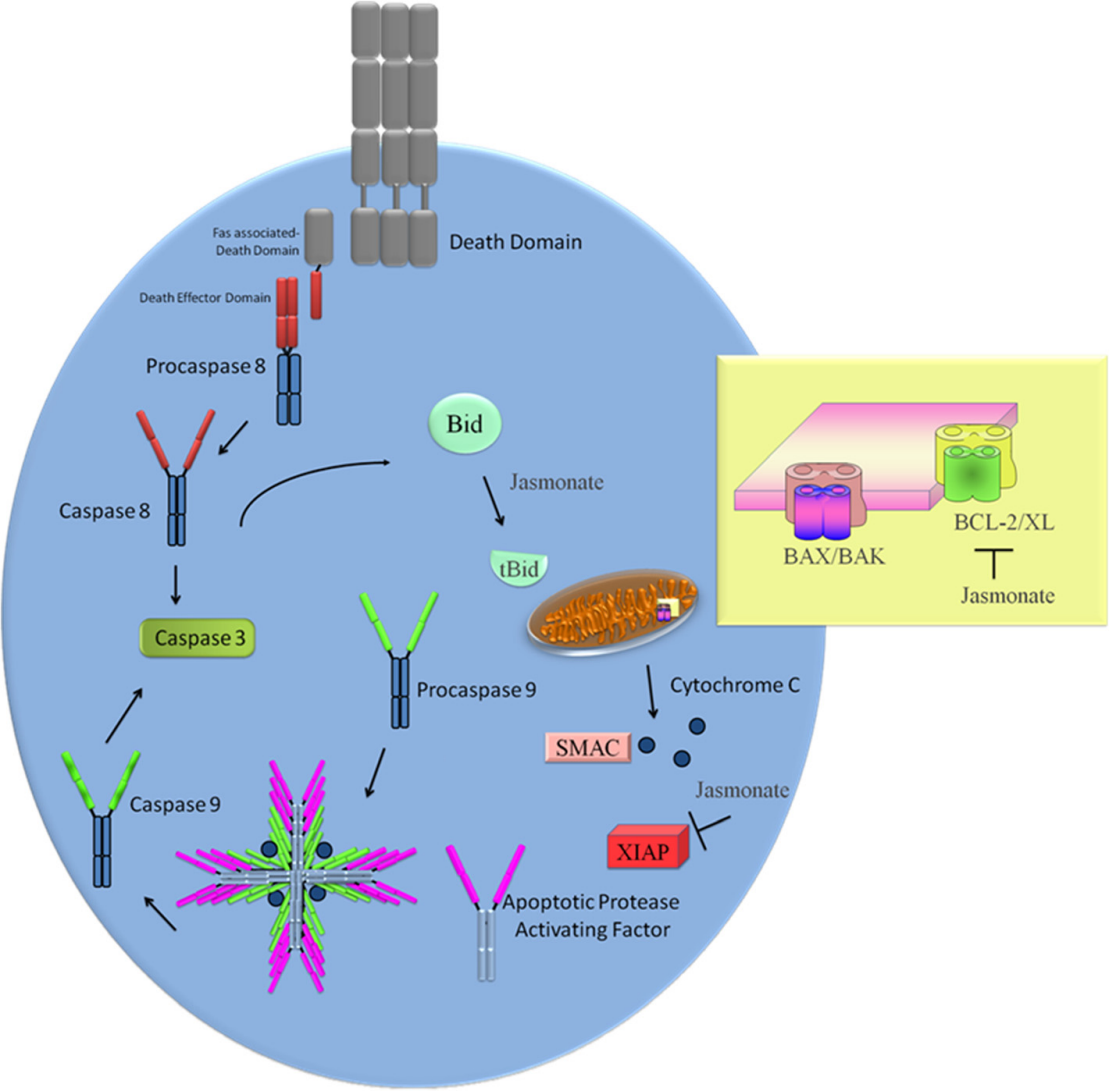
**Showing TRAIL mediated apoptosis.** There is an overexpression of antiapoptotic proteins that impairs the cell death. Jasmonates induce apoptosis via multistep activity.

Tumour necrosis factor-related apoptosis-inducing ligand (TRAIL) has emerged as a potent anticancer agent in laboratory-based studies and preclinical trials. In this forth coming section we discuss, the experimental evidence that supports a role for jasmonates in sensitizing cancer cells to TRAIL mediated apoptosis. Adding together different pieces of information indicate that although TRAIL is able to induce apoptosis in prostate cancer cell, yet overexpression of antiapoptotic proteins and inhibition of pro-apoptotic proteins results in dampening the TRAIL mediated apoptosis. Another important feature is that the receptors (death receptors) of TRAIL are outnumbered by decoy receptors that further confound TRAIL mediated therapy (Figure 1).

MJ is documented to be involved in suppressing the negative regulators of TRAIL mediated apoptosis. In line with this approach a study demonstrated that survivin negatively regulated TRAIL mediated apoptosis. Astonishingly, MJ-induced TRAIL sensitization was observed and overexpression of survivin prevented MJ-induced TRAIL cytotoxicity (Raviv et al., [36]). On a similar note, J7, a novel MJ analogue enhances TRAIL-induced apoptosis. The apoptosis was induced via Bid cleavage, down-regulation of XIAP, cIAP-1 and Bcl-xL and activation of caspases (Park et al., [37]) (Figure 1).

It has been shown extensively previously that mixtures of interacting compounds underscore vital combination therapies that concurrently hit effectively multiple pharmacological targets and provide effectiveness beyond the reach of single compound–based drugs. In accordance with the multiple properties of algae derived components and methyl jasmonates, development of innovative scientific methods for discovery, validation, characterization and standardization of these multicomponent therapeutics is essential to their acceptance into mainstream medicine. Therefore it is indispensable that attention needs to be focused on intracellular-signalling cascades as common molecular targets for these agents.

## Conclusion

With incredible progress in exploring the efficacy of herbal extracts as potent anticancer agents various limitations do exist. It is important to have characterization of bioactive components as various components having opposite effects on the cells impair the efficacy when combined with the agents believed to induce apoptosis. In line with this approach, a recent study indicates that aqueous extracts of *Gracilaria tenuistipitata* are documented to rescue cells from undergoing apoptosis (Yang et al., [38]). Contrary to this metahnolic/ethanolic extracts were proved to be potent anticancer agents. Ca9-22 oral cancer cell line was tested for efficacy of Methanolic extracts of *Gracilaria tenuistipitata* and results indicated that the extracts induced apoptosis in cancer cells via enhanced ROS production and DNA damage (Yeh et al., [39]). Interestingly, Ethanolic Extracts of *Gracilaria tenuistipitata* were also found to be effective in concentration dependent manner in oral cancer cells (Yeh et al., [40]).

Accumulating data offers new insights in the study of alternative therapeutic strategies in the treatment of prostate cancer. Further consideration deserves to be given to the differences in the cellular biology of sensitive tumours that could explain the cytotoxic effect of algae derived ingredients and the increase in the cytotoxic response caused by these biomolecules, in particular cancer types. Our laboratory is focusing and studying biological activities of various extracts derived from local strains of algae (Ghazala et al., [41]; Ghazala et al., [42]; Ghazala et al., [42]; Ghazala and Shameel, [43]; Ghazala et al., [44]; Ghazala et al., [45]; Ghazala et al., [46]; Ghazala et al.,[47]). Additionally anticancer potential of these extracts is currently being tested to identify agents which could improve the efficacy of mechanism-based and targeted therapeutic strategies against cancer.

It is appealing to note that prevention of prostate cancer could occur in a multipronged manner. These approaches include counteracting uncontrolled cellular proliferation, restoration of cell death pathway, and repression of the factors that underlie genesis of genomic rearrangements in prostate cancer cells.

No published research has addressed the effects of algae derived components or methyl jasmonates on intraprostatic sex-steroid levels. Additionally, how these ingredients influence the expression and activity of the androgen receptor (a key mediator of the effects of androgens) is also unknown and direction of future clinical trials lies in clarifying the effects of these agents and exploring the biological mechanisms responsible for the prevention of prostate cancer. Furthermore, there are various signaling mechanisms that work synchronously and facilitate escape of cells from apoptosis Farooqi et al., [48]. In addition there are miRNA’s which are categorized into oncomirs and tumor suppressors and are documented to be involved in regulation of apoptosis are also attractive targets. A recent study indicated that pomegranate juice stimulated the expression of tumor suppressor miRNA’s and downregulated oncomirs in prostate cancer Wang et al., [49]. Likely, mechanisms that underpin initiation and progression of prostate cancer so far proposed include AR mutations, allowing receptors to be activated by new ligands. AR gene overexpression causing AR hypersensitivity to declining concentrations of androgen and cross-stimulation of AR signaling by other growth factors and how AR transmigration into nucleus to modulate transcriptional network are some of the prominent features that need a detailed investigation with reference to methyl jasmonates and algae derived components.

It is worthwhile that latest biology and high-tech genomics research are helping us in devising treatments that target stratified proteome of cancer cell. Keeping in view cellular plasticity and cancer heterogeneity, therapeutic interventions are less effective with threatening off target effects. Moreover, many anticancer drugs work by killing all the dividing cells — not just the cancerous ones but healthy cells in addition.

## Competing interests

The authors declare that they have no conflict of interest.

## Authors’ contributions

AAF, GB and ZR drafted different portions of the manuscript. AAF designed the figure. All authors have reviewed the manuscript and agreed with its contents.
